# The characteristics of impaired fasting glucose associated with obesity and dyslipidaemia in a Chinese population

**DOI:** 10.1186/1471-2458-10-139

**Published:** 2010-03-17

**Authors:** Yun Qian, Yudi Lin, Tiemei Zhang, Jianling Bai, Feng Chen, Yi Zhang, Senlin Luo, Hongbing Shen

**Affiliations:** 1 Department of Disease Control and Prevention, Wuxi Center for Disease Prevention and Control, Wuxi 214023, Jiangsu, China; 2 Department of Epidemiology and Biostatistics, Nanjing Medical University School of Public Health, Nanjing 210029, Jiangsu, China; 3 Department of Epidemiology, Institute of Geriatrics of Beijing Hospital, Ministry of Health, Beijing 100730, China; 4 Department of Information, Beijing Institute of Technology, Beijing 100081, China

## Abstract

**Background:**

Different populations have diverse patterns of relationships between Impaired Fasting Glucose (IFG) and obesity and lipid markers, it is important to investigate the characteristics of associations between IFG and other related risk factors including body mass index (BMI), waist circumstance (WC), serum lipids and blood pressure (BP) in a Chinese population.

**Methods:**

This was a case-control study of 648 IFG subjects and 1,296 controls derived from a large-scale, community-based, cross-sectional survey of 10,867 participants. Each subject received a face-to-face interview, physical examination, and blood tests, including fasting blood glucose and lipids. Student's *t*-test, Chi-square test, Spearman correlation and multiple logistic regressions were used for the statistical analyses.

**Results:**

Fasting plasma glucose (FPG) was positively correlated with BMI, WC, systolic blood pressure (SBP), diastolic blood pressure (DBP), triglyceride (TG), and total cholesterol (TC), and was negatively correlated with high density lipoprotein-cholesterol (HDL-C) (all p < 0.05). BMI was more strongly correlated with IFG than with WC. The correlation coefficient of FPG was remarkably higher with TG (0.244) than with TC (0.134) and HDL-C (-0.192). TG was an important predictor of IFG, with odds ratios of 1.76 (95%CI: 1.31-2.36) for subjects with borderline high TG level (1.70 mmol/l ≤ TG < 2.26 mmol/l) and 3.13 (95% CI: 2.50-3.91) for those with higher TG level (TG ≥ 2.26 mmol/l), when comparing to subjects with TG < 1.70 mmol/l. There was a significant dose-response relationship between the number of abnormal variables and increased risk of IFG.

**Conclusions:**

In this Chinese population, both BMI and WC were important predictors of IFG. Abnormal TG as a lipid marker was more strongly associated with IFG than were TC and HDL-C. These factors should be taken into consideration simultaneously for prevention of IFG.

## Background

Impaired fasting glucose (IFG) is a frequent glycemic disorder in the general population and is considered as a pre-diabetic state [1]. IFG has received increasing attention in recent years, not only because it is an intermediate stage in the development of diabetes and cardiovascular diseases (CVDs) [2-4], but also because it is associated with increased risk of all-cause death and CVD mortality [5]. IFG has thus come to be considered as a potential indicator of preventive importance for diabetes and CVDs [6].

IFG was first introduced as a concept by the American Diabetes Association (ADA) in 1997 and was defined as the presence of abnormal fasting plasma glucose (FPG ≥ 6.1 mmol/l and < 7.0 mmol/l) [7]. Nevertheless, in the light of evidence from several epidemiological studies, the ADA lowered the threshold for diagnosis of IFG to FPG ≥ 5.6 mmol/l, with the expectation that progression of pre-diabetes into diabetes and the development of CVD can be prevented through earlier awareness and intervention [8]. IFG occurs frequently in Chinese populations, with a prevalence of 21.6% among adults age 35-64 years [9], as defined by the new criterion of ≥ 5.6 mmol/l and < 7.0 mmol/l [8].

In addition to IFG, other common risk factors associated with diabetes and CVD, such as obesity and dyslipidaemia, merit consideration for the prevention of non-communicable diseases. Because different populations have diverse patterns of relationships between IFG and obesity and lipid markers, it is important to investigate the characteristics of associations between IFG and other related risk factors in the Chinese population. In addition, because IFG is the early stage of diabetes and cardiovascular diseases, identifying preventable risk factors associated with IFG at this early stage is very important in prevention and control of these diseases. Therefore, in this study we present the relationships between IFG and obesity and lipid markers ascertained in a community-based case-control study of IFG in Wuxi city, south Jiangsu Province, China.

## Methods

This study was approved by the ethnic committee of the Institute of Geriatrics of Beijing Hospital, Ministry of Health of China. The case-control study of IFG was derived from a large-scale, community-based, cross-sectional survey of non-communicable diseases conducted in the Wuxi municipality, Jiangsu Province of eastern China, from April to July 2007. All participants in this cross-sectional survey were permanent local residents aged more than 20 years old, and all had signed informed consent documents. A total of 10,867 subjects completed an interview and physical examination and all of them were ethnic Han Chinese. We selected as cases of IFG all subjects whose FPG was between 5.6 mmol/l and 7.0 mmol/l (2004 ADA definition) after fasting more than 8 hours. Subjects who self-reported a history of diabetes (7.40%) and other chronic diseases (36.42%, e.g. hypertension, coronary heart diseases, dyslipidaemia and stroke) were excluded. Two healthy controls who did not report any chronic diseases and had an FPG < 5.6 mmol/l were randomly chosen from the database for each case, matched on age (± 5 years), gender and resident district, by using SAS matching MACRO. As a result, 648 IFG cases and 1296 age- and sex-matched controls were available for the analyses.

All participants were interviewed by trained interviewers using standard questionnaires to obtain information on demographic data, smoking, alcohol drinking, and family history of diabetes and CVDs in first-degree relatives (parents, siblings and children). Height and weight were measured to within 0.5 cm and 0.1 kg, respectively. Body mass index (BMI) (kg/m^2^) was used as an indicator of overall adiposity. Waist circumference (WC), a validated estimate of abdominal adiposity, was measured to within 0.5 cm. Sitting blood pressure (systolic and fifth-phase diastolic) was recorded 3 times with a standard mercury sphygmomanometer after a 15-min rest.

A 5 ml blood sample was collected from each subject after fasting more than 8 hours. The coagulated blood was then centrifuged at 3,000 rpm for 10 min. The serum was used to measure glucose levels and lipid markers, including total cholesterol (TC), triglyceride (TG), and high-density lipoprotein-cholesterol (HDL-C), within 2 hours after blood collection. All the biochemical markers were measured using an OLYMPUS (C2734-Au640) automatic analyzer in the central laboratory of Wuxi Center for Disease Prevention and Control, which was authorized to perform laboratory tests according to the international quality standard ISO/IEC 17025. The Beijing Institute of Geriatrics of Beijing Hospital, Ministry of Health performed regular periodic assessments of laboratory quality using quality control samples.

IFG was defined according to the 2004 ADA definition as FPG from 5.6 mmol/l to 7.0 mmol/l. The definition of dyslipidaemias accorded with the criteria of the "China Guidelines on Prevention and Management of Dyslipidaemias in Chinese Adults."[10] The borderline high cholesterol level was 5.18-6.21 mmol/l, and subjects were defined as having hypercholesterolemia if their TC was ≥ 6.22 mmol/l. The borderline high triglyceride level was 1.70-2.25 mmol/l, and subjects were defined as having high triglyceride if their TG were ≥ 2.26 mmol/l. Subjects were defined as having low HDL-C if their HDL-C levels were < 1.04 mmol/l. BMI was calculated as weight (kg)/height (m)^2^, and subjects were considered as normal weight if their BMI was < 24 kg/m^2^, overweight if their BMI was from 24 to 27.9 kg/m^2 ^and obese if their BMI was ≥ 28 kg/m^2^. Subjects were considered as having central obesity if their WC was > 85 cm for men and > 80 cm for women [11]. Those whose average systolic blood pressure (SBP) was ≥ 140 mmHg and/or diastolic blood pressure (DBP) ≥ 90 mmHg were defined as hypertensive, according to the WHO criteria.

We used SAS 9.1.3 (SAS Institute, Inc., Cary, NC) for data management and analyses. Means and their standard deviations (SDs) were used for the continuous variables, and frequencies and percentages were used for categorical variables in the analysis. Spearman correlation analysis was used to estimate the relationships among related variables. Odds ratios (ORs) and their 95% confidence intervals (95%CIs) from the multivariate logistic regression models were used to evaluate associations between IFG and other risk factors, after adjusting the available covariates such as education level, monthly income and family history of diabetes. We also assessed the joint dose-response effects of overweight, hypertension and dyslipidemia on the odds of IFG by defining as abnormal a BMI ≥ 24 kg/m^2^, a SBP ≥ 140 mmHg and/or DBP ≥ 90 mmHg, a TG ≥ 1.70 mmol/l, a HDL-C < 1.04 mmol/l, and a TC ≥ 5.18 mmol/l, and evaluated the risk of IFG associated with the number of abnormalities. All statistical tests were two-sided, and p-values ≤ 0.05 were considered statistically significant.

## Results

The total number of subjects included in this analysis was 1944, of whom 648 were cases of IFG and 1296 were matched controls with normal blood glucose. Of the 1944 subjects, 45.06% were males and 54.94% were females. Their ages ranged from 21 years to 79 years. The means and standard deviations (SDs) of age were 60.53 ± 11.39 years for IFG cases and 59.75 ± 11.29 years for controls, respectively. There were no statistically significant differences in age, gender, educational level and individual monthly income between IFG subjects and controls (Table 1). More IFG subjects (10.56%) reported having family history of diabetes than controls (6.45%) (p = 0.002, Table 1). As expected, IFG subjects had significantly higher BMI, WC, TC, TG, SBP and DBP, and lower HDL-C than did controls, and these differences were seen in both men and women. (Table 2)

**Table 1:** Social-demographic characteristics of IFG subjects and controls in a Chinese population

Variables	IFG (n = 648) No. (%)	controls (n = 1296) No. (%)	p value
Gender			1.000
Male	292 (45.06)	584 (45.06)	
Female	356 (54.94)	712 (54.94)	
Age (years) (mean ± SD)	60.53 ± 11.39	59.75 ± 11.29	0.151
Age (years)			0.253
<45	54 (8.34)	122 (9.41)	
45~60	232 (35.80)	501 (38.66)	
≥ 60	362 (55.86)	673 (51.93)	
Education level			0.051
Illiteracy or primary school	190 (29.32)	333 (25.70)	
Middle and high school	354 (55.05)	704 (54.62)	
College and higher	104 (16.05)	259 (20.09)	
Individual monthly income (RMB) *			0.090
< 1000	300 (49.42)	532 (44.22)	
1000~2000	280 (46.13)	603 (50.13)	
≥ 2000	27 (4.45)	68 (5.65)	
Family history of diabetes**			0.002
No	576 (89.44)	1203 (93.55)	
Yes	68 (10.56)	83 (6.45)	

* 134 subjects (41 cases and 93 controls) did not provide the individual monthly income information

** 14 subjects (4 cases and 10 controls) did not provide the family history of diabetes information

**Table 2:** Comparison of the levels of BMI, WC, BP and lipids between IFG subjects and controls

Variables	Overall		Men		Women	
			
	IFG (n = 648)	Control (n = 1296)	IFG (n = 292)	Control (n = 584)	IFG (n = 356)	Control (n = 712)
BMI(kg/m^2^)	25.34 ± 3.44	23.21 ± 3.11**	25.32 ± 3.15	23.27 ± 2.94**	25.36 ± 3.67	23.16 ± 3.25**
WC(cm)	89.21 ± 10.10	83.92 ± 9.92**	90.93 ± 9.72	85.76 ± 9.14**	87.82 ± 10.20	82.42 ± 10.28**
TG(mmol/l)	2.98 ± 2.55	2.05 ± 1.69**	2.98 ± 2.61	2.06 ± 1.77**	3.00 ± 2.49	2.05 ± 1.62**
HDL-C(mmol/l)	1.28 ± 0.31	1.41 ± 0.37**	1.22 ± 0.30	1.32 ± 0.34**	1.33 ± 0.32	1.49 ± 0.38**
T C (mmol/l)	5.06 ± 1.09	4.84 ± 0.93**	4.93 ± 1.05	4.66 ± 0.85**	5.16 ± 1.11	4.99 ± 0.98*
SBP(mmHg)	134.22 ± 19.05	126.07 ± 17.26**	130.07 ± 19.85	128.22 ± 17.50**	132.70 ± 18.25	124.32 ± 16.88**
DBP(mmHg)	83.20 ± 10.23	79.31 ± 9.43**	84.73 ± 10.61	81.05 ± 9.54**	81.95 ± 9.74	77.89 ± 9.10**

*p < 0.05; ** p < 0.01

In the bivariate Spearmen correlation analyses (Table 3), FPG was positively correlated with BMI, WC, SBP, DBP, TG, TC, and negatively correlated with HDL-C. The highest correlation coefficients (r) with FPG were 0.325 for BMI and 0.266 for WC. For the three lipid markers, TG was more strongly correlated with FPG (r = 0.244) than TC (r = 0.134) and HDL-C (r = -0.192). In the logistic regression analyses, with adjustment for education level, individual monthly income, family history of diabetes (Table 4), BMI was strongly associated with increased risk of IFG in both men and women. Overweight (24 kg/m^2 ^≤ BMI < 28 kg/m^2^) and obesity (BMI ≥ 28 kg/m^2^) were associated with a 2.32-fold (OR = 2.32, 95%CI: 1.86-2.88) and a 4.63-fold (OR = 4.63, 95%CI: 3.36-6.37) higher risk of IFG, respectively, compared with normal-weight subjects. The effect of obesity (BMI ≥ 28 kg/m^2^) was more evident in male subjects (OR = 6.37, 95%CI: 3.76-10.79). Central obesity (WC ≥ 85 cm in males and WC ≥ 80 cm in females) was associated with a 2.74-fold elevated risk (OR = 2.74, 95%CI: 2.19-3.44) of IFG relative to those with normal WC. For the lipid markers, both TC and TG were significantly associated with IFG. Nevertheless, TG appeared to be more strongly associated with IFG than TC in this population. Compared to subjects with TG < 1.70 mmol/l, those with borderline high TG levels (1.70 to 2.26 mmol/l) and high TG (≥ 2.26 mmol/l) had a 1.76-fold (95%CI: 1.31-2.36) and a 3.13-fold (95%CI: 2.50-3.91) elevated risk of IFG, respectively. Compared to the normal TC level group (TC < 5.18 mmol/l), the odds of IFG were significantly higher in persons with higher TC levels (OR = 1.32, 95%CI: 1.06-1.66 for those with 5.18 mmol/l ≤ TC < 6.22 mmol/l; and OR = 2.01, 95%CI: 1.42-2.84 for those with TC ≥ 6.22 mmol/l). Higher HDL-C was significantly associated with a decreased risk of IFG (OR = 0.43, 95%CI: 0.34-0.56). In addition, hypertension was associated with a 2.68-fold (95%CI: 2.19-3.29) increased odds of IFG compared to those with normal blood pressure.

**Table 3:** Spearman correlation analysis of selected variables in both IFG subjects and controls (n = 1944)

r		WC	SBP	DBP	TG	HDL-C	TC	FPG
BMI	overall	0.701**	0.218**	0.248**	0.248**	-0.298**	0.083**	0.325**
	men	0.710**	0.214*	0.252**	0.267**	-0.322**	0.123**	0.340**
	women	0.707**	0.223**	0.249**	0.176**	-0.290**	0.060	0.315**
WC	overall		0.267**	0.251**	0.209**	-0.282**	0.095**	0.266**
	men		0.243**	0.240**	0.252**	-0.244**	0.178**	0.288**
	women		0.266**	0.227**	0.180**	-0.266**	0.084**	0.262**
SBP	overall			0.711**	0.128**	-0.068**	0.099**	0.248**
	men			0.738**	0.084*	0.056	0.075*	0.225**
	women			0.678**	0.170**	-0.129**	0.147**	0.276**
DBP	overall				0.157**	-0.117**	0.084**	0.214**
	men				0.155**	-0.023	0.097**	0.200**
	women				0.162**	-0.138**	0.120**	0.236**
TG	overall					-0.431**	0.300**	0.244**
	men					-0.413**	0.328**	0.218**
	women					-0.464**	0.285**	0.268**
HDL-C	overall						0.249**	-0.192**
	men						0.191**	-0.145**
	women						0.247**	-0.244**
TC	overall							0.134**
	men							0.143**
	women							0.124**

*p < 0.05; ** p < 0.01

**Table 4:** Associations between BMI, WC, blood lipids, BP and risk of IFG

Variables	IFG(%) (n = 648)	Control(%) (n = 1296)	Adjusted-OR* (95%CI)(overall)	Adjusted-OR* (95%CI)(men)	Adjusted-OR* (95%CI)(women)
**BMI (kg/m^2^)**					
BMI < 24	233 (35.95)	793 (61.19)	1.00	1.00	1.00
24 ≤ BMI < 28	286 (44.14)	413 (31.87)	2.32 (1.86-2.88)	2.23 (1.61-3.09)	2.43 (1.79-3.28)
BM I ≥ 28	129 (19.91)	90 (6.94)	4.63 (3.36-6.37)	6.37 (3.76-10.79)	3.98 (2.64-6.00)
**WC (cm)**					
WC < 85 (male) WC < 80 (female)	140 (21.60)	562 (43.36)	1.00	1.00	1.00
WC ≥ 85 (male) WC ≥ 80 (female)	508 (78.40)	734 (56.64)	2.74 (2.19-3.44)	2.57 (1.85-3.56)	3.00 (2.18-4.13)
**TC (mmol/l)**					
TC < 5.18	382 (58.95)	878 (67.75)	1.00	1.00	1.00
5.18 ≤ TC < 6.22	192 (29.63)	329 (25.39)	1.32 (1.06-1.66)	1.53 (1.08-2.17)	1.22 (0.90-1.64)
TC ≥ 6.22	74 (11.42)	89 (6.86)	2.01 (1.42-2.84)	2.47 (1.34-4.57)	1.86 (1.21-2.84)
**TG (mmol/l)**					
TG < 1.70	203 (31.33)	696 (53.70)	1.00	1.00	1.00
1.70 ≤ TG < 2.26	107 (16.51)	213 (16.44)	1.76 (1.31-2.36)	2.18 (1.40-3.40)	1.51 (1.02-2.25)
TG ≥ 2.26	338 (52.16)	387 (29.86)	3.13 (2.50-3.91)	3.09 (2.20-4.33)	3.26 (2.40-4.43)
**HDL-C (mmol/l)**					
HDL-C < 1.04	153 (23.61)	160 (12.35)	1.00	1.00	1.00
HDL-C ≥ 1.04	495 (76.39)	1136 (87.65)	0.43 (0.34-0.56)	0.44 (0.31-0.63)	0.42 (0.29-0.62)
**BP (mmHg)**					
SBP < 140 and DBP < 90	308 (47.53)	901 (69.52)	1.00	1.00	1.00
SBP ≥ 140 and/or DBP ≥ 90	340 (52.47)	395 (30.48)	2.68 (2.19-3.29)	2.32 (1.71-3.14)	3.05 (2.30-4.04)

* adjusted for education level, individual monthly income, family history of diabetes.

**Figure 1:**
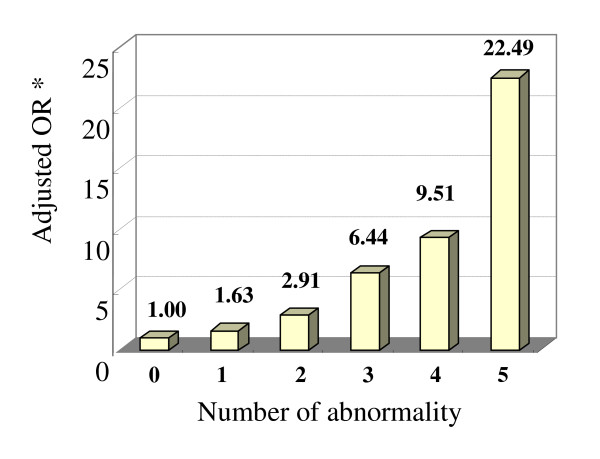
**Combined effect of 5 abnormal variables on the risk of IFG**. (5 abnormal variables include BMI ≥ 24 kg/m^2^, SBP ≥ 140 mmHg and/or DBP ≥ 90 mmHg, TG ≥ 1.70 mmol/l, HDL-C < 1.04 mmol/l, and TC ≥ 5.18 mmol/l) (* adjusted for education level, monthly income and family history of diabetes).

We also assessed the joint effects of overweight (BMI ≥ 24 kg/m^2^), hypertension (SBP ≥ 140 mmHg and/or DBP ≥ 90 mmHg) and dyslipidemia (TG ≥ 1.70 mmol/l, HDL-C < 1.04 mmol/l and/or TC ≥ 5.18 mmol/l) on the odds of IFG. We evaluated the risk of IFG associated with the number of above 5 abnormalities. There was a significant dose-response relationship between an increased number of abnormalities and the odds of IFG (p for trend < 0.001). Subjects having 1, 2, 3, 4 and 5 abnormal items had 1.63 (95%CI: 1.08-2.46), 2.91 (95%CI: 1.98-4.28), 6.44 (95%CI: 4.37-9.49), 9.51 (95%CI: 6.13-14.73) and 22.49 (95%CI: 7.03-71.96) times higher odds of IFG compared to those without abnormalities, respectively (Figure 1).

When we took the joint effects of BMI and WC into consideration, we found that subjects with both abnormal BMI (≥ 24 kg/m^2^) and WC (≥ 85 cm for men and ≥ 80 cm for women) had a statistically significant 4.06-fold (95%CI: 3.12-5.28) elevated odds for IFG, almost double that of subjects with only one abnormal variable (OR = 2.09, 95%CI: 1.54-2.85) for abnormal WC and OR = 2.59, 95%CI: 1.61-4.17 for higher BMI, respectively) (Table 5). These associations were similar for both men and women. For the combination of TG and TC, subjects with both abnormal TG (≥ 1.70 mmol/l) and TC (≥ 5.18 mmol/l) had an odds ratio of 3.03 (95%CI: 2.31-3.97), while those with elevated TG alone had an OR of 2.97 (95%CI: 2.28-3.85), and those with elevated TC alone had an OR of 1.63 (95%CI: 1.13-2.35), indicating that TG was the more important contributor to the association (Table 5).

**Table 5:** Combined effects of BMI and WC, TG and TG associated with IFG

Combined variables	IFG(%) (n = 648)	Control(%) (n = 1296)	Adjusted-OR* (95%CI)(overall)	Adjusted-OR* (95%CI)(men)	Adjusted-OR* (95%CI)(women)
**BMI and WC**					
BMI < 24 and WC < 85(men) 80(women)	106 (16.36)	488 (37.65)	1.00	1.00	1.00
BMI < 24 and WC ≥ 85(men) 80(women)	127 (19.60)	305 (23.53)	2.09 (1.54-2.85)	1.83 (1.14-2.95)	2.39 (1.59-3.61)
BMI ≥ 24 and WC < 85(men) 80(women)	34 (5.24)	74 (5.71)	2.59 (1.61-4.17)	2.26 (1.19-4.29)	3.00 (1.48-6.11)
BMI ≥ 24 and WC ≥ 85(men) 80(women)	381 (58.80)	429 (33.10)	4.06 (3.12-5.28)	3.72 (2.54-5.44)	4.54 (3.13-6.57)
**TG and TC**					
TG < 1.70 and TC < 5.18	143 (22.07)	550 (42.44)	1.00	1.00	1.00
TG < 1.70 and TC ≥ 5.18	60 (9.26)	146 (11.26)	1.63 (1.13-2.35)	2.82 (1.56-5.08)	1.22 (0.75-1.97)
TG ≥ 1.70 and TC < 5.18	239 (36.88)	328 (25.31)	2.97 (2.28-3.85)	3.50 (2.39-5.12)	2.62 (1.82-3.77)
TG ≥ 1.70 and TC ≥ 5.18	206 (31.79)	272 (20.99)	3.03 (2.31-3.97)	3.39 (2.22-5.18)	2.84 (1.96-4.08)

* adjusted for education level, individual monthly income, family history of diabetes.

## Discussion

In this community-based, case-control study of impaired fasting glucose in a Chinese population, we confirmed previous reports of significant associations between fasting plasma glucose and body mass index, waist circumference, total cholesterol, triglycerides, systolic and diastolic blood pressure, and HDL-cholesterol. Levels of BMI, WC, TG, TC, SBP and DBP were significantly higher in cases with IFG than in normoglycemic controls, whereas the level of HDL-C was significantly lower in both men and women with IFG. Both BMI and WC were significantly related to FPG, TG, TC, HDL-C, SBP and DBP. The correlation between BMI and FPG was greater than that for WC and FPG. TG was more strongly correlated with FPG than TC and HDL-C. The odds of IFG increased with the numbers of abnormal variables.

Our finding of significantly positive relationships between IFG and BMI and WC are consistent with reports from other studies in different populations [12,13] and indicate that overweight and obesity could result in higher insulin concentration, secretion and resistance [14]. Hu *et al*., found that central obesity was more related to IFG than overall obesity in a northern Chinese population [15]. However, in our study BMI was more strongly related to FPG than WC, as indicated by the higher correlation coefficient, and we found that subjects with both central obesity and overall obesity had a doubled odds of IFG compared to those with only one of these abnormalities. This finding suggests that both BMI and WC should be taken into consideration as obesity indicators that predict IFG in the Chinese population, and they should be considered simultaneously as targets for possible interventions to prevent IFG.

Consistent with other studies [16,17], our present study indicated that TG and TC levels were positively correlated with FPG and that HDL-C was negatively correlated with FPG in both male and female populations. The dysregulation of lipid and lipoprotein in the hyperglycaemic state may be a manifestation of underlying insulin resistance, which is a central pathophysiological feature of type 2 diabetes and abdominal obesity. Insulin resistance results in hyperinsulinemia, enhanced hepatic gluconeogenesis and glucose output. It also reduces suppression of lipolysis in adipose tissue, leading to high free acid flux, and it increases hepatic very low density lipoprotein secretion leading to hypertriglyceridemia and reduced levels of HDL-C [18]. However, many studies have shown that dysregulation of fatty acid metabolism plays the key role in the etiology of IFG [19,20]. An important observation from the San Antonio Heart Study was that several independent predictors of diabetes that were present before the onset of hyperglycaemia were correlated with each other, including elevated BMI and insulin levels, hypertriglyceridaemia, hypertension and lower HDL-C [21]. Obesity was also one of the most important factors associated with serum lipid profiles in our study, in which both BMI and WC were positively correlated with TG and TC and negatively correlated with HDL-C. These factors of obesity and abnormal lipids might work together and reinforce the development of IFG. Our study results also suggest that TG may be more strongly correlated with IFG than TC and HDL-C. Love-Osborne and colleagues reported that elevated fasting triglycerides could predict risk of impaired glucose tolerance [22]. TG, as a lipid marker, also can be used as one of the most important predictors of IFG.

There was also a significant association between high blood pressure and increased blood glucose in our study. The association between hypertension and IFG can probably be related to the metabolic syndrome [23], and a modest association between hyperinsulinaemia and hypertension has been reported previously [24]. In a cohort of 63,443 consecutive men, Patrick, *et al*., demonstrated that risk ratios for 8-year cardiovascular disease mortality and total mortality were significantly higher in the IFG subjects with 140 ≤ SBP ≤ 159 mmHg compared to those with normal fasting glucose [25]. The close relationship between insulin resistance and hypertension may be because of their similar molecular mechanisms [26,27].

All the risk factors in our study, including overweight (obesity), dyslipidaemias and hypertension are components of the metabolic syndrome and insulin resistance. The World Health Organization (WHO) working definition of metabolic syndrome includes a raised plasma TG and/or low HDL-C, central obesity and/or high BMI ratio, measurement of impaired glucose regulation or/and insulin resistance, arterial hypertension and microalbuminuria [28]. In the present study of this Chinese population, we found that elevated TG, both central (WC) and overall obesity (BMI), and hypertension were important predictors of IFG, which is a key representation of the metabolic syndrome [29]. Rapid economic development and changing lifestyles in the Chinese population in the past three decades have resulted in a steady increase in the prevalence of obesity [30]. The strong association between overweight and IFG emphasizes the importance of weight control interventions to reduce the prevalence of IFG in the population. Identifying and reducing these factors, including BMI, WC, TG, and BP, as well as FPG, may improve the health of the general population of China, and more programs should be targeted towards control of IFG and related public health problems.

Several limitations of this study need to be addressed. Firstly, this is a cross-sectional investigation and it is really difficult to determine which is the risk factor and which is the outcome in terms of the variables included in this study. Secondly, all of our IFG cases were from one community of Wuxi city, a rapidly developed area in eastern China, which may not represent for those of other places. Large multi-center prospective follow-up studies or intervention studies are warranted to further evaluate the public health meanings of these risk factors.

## Conclusions

In this Chinese population, both BMI and WC were important predictors of IFG. Abnormal TG as a lipid marker was more strongly associated with IFG than were TC and HDL-C. These factors should be taken into consideration simultaneously for prevention of IFG.

## Abbreviations

IFG: impaired fasting glucose; BMI: body mass index; WC: waist circumstance; BP: blood pressure; SBP: systolic blood pressure; DBP: diastolic blood pressure; FPG: fasting plasma glucose; TG: triglyceride; TC: total cholesterol; HDL-C: high density lipoprotein-cholesterol; CVD: cardiovascular disease; OR: odds ratio; CI: confidence interval.

## Competing interests

The authors declare that they have no competing interests.

## Authors' contributions

YQ carried out the field study, performed the statistical analysis and drafted the manuscript. YL, TZ, FC and HS participated in the study design and coordination and helped to refine the manuscript. JB, YZ, SL participated in the field investigation. All authors read and approved the final manuscript.

## Pre-publication history

The pre-publication history for this paper can be accessed here:

http://www.biomedcentral.com/1471-2458/10/139/prepub
